# The positive role of authentic leadership in organizations negatively affected by cognitive diversity

**DOI:** 10.3389/fpsyg.2024.1276585

**Published:** 2024-04-25

**Authors:** Kaori Yagi, Junko Iida, Kei Fuji

**Affiliations:** ^1^Pallas Athena Inc., Tokyo, Japan; ^2^Faculty of Human Sciences, University of Tsukuba, Tsukuba, Japan

**Keywords:** cognitive diversity, authentic leadership, information elaboration, team process, multilevel SEM

## Abstract

Workplace diversity has recently gained increasing significance and urgency in business organizations. This promotion may stem from information processing, and specifically from information elaboration. Information elaboration leverages diverse task-related information and skills possessed by members, fostering the exchange of diverse perspectives, elaborate discussions, and achieving high team performance. In this context, cognitive diversity, encompassing members’ knowledge, skills, and perspectives, may have a positive impact. However, some previous studies suggest that cognitive diversity can lead to affective conflict and impede information processing. In organizations with highly homogeneous social and cultural backgrounds, cognitive diversity may not be effectively utilized in the information elaboration process, potentially yielding negative effects. Authentic leadership is recognized as a significant contributor to facilitating team processes including information processing, with various studies demonstrating its effectiveness. This study hypothesized that cognitive diversity negatively affects the information elaboration process, while authentic leadership has a positive effect. To test these hypotheses, we employed multilevel structural equation modeling analysis based on data collected from 375 respondents in 90 teams across various industries in Japan. The results showed that cognitive diversity negatively affects information elaboration at the individual level. By contrast, authentic leadership positively affects information elaboration at both individual and team levels. These findings suggest that the effect of cognitive diversity on information processing in the workplace may not always be positive, particularly in a sociocultural context that values homogeneity, as observed in Japanese organizations. This study advances the literature on authentic leadership by validating its effect on information elaboration and provides practical implications for diversity management. Additionally, it underscores the effectiveness of authentic leadership in leveraging team members’ cognitive diversity to facilitate information elaboration.

## 1 Introduction

Addressing workplace diversity is a crucial concern for business organizations in today’s highly dynamic and globalized environment. Diversity encompasses differences among individuals based on various attributes, both visible and invisible, that can lead to diverse perceptions, potentially impacting team outcomes positively or negatively (van Knippenberg et al., 2004).

Regarding the positive impact, team members’ diverse knowledge and information, spanning opinions, ideas, perspectives, insights, and values, can greatly benefit information processing within a team. This can result in high-quality decision-making, effective problem-solving, enhanced creativity, and innovation (van Knippenberg et al., 2004). Information processing, defined as elaborating task-relevant information and perspectives (information elaboration; IE) (van Knippenberg et al., 2004), has been established as effective in various business organizations across performance parameters (e.g., Kearney and Gebert, 2009; Lee and Yang, 2015; Oad and Niu, 2017; Maynard et al., 2019; Wei et al., 2019).

Cognitive diversity, focusing on differences in individuals’ invisible attributes such as beliefs, preferences, attitudes, values, worldview, perspectives, knowledge, and skills (Miller et al., 1998; Kilduff et al., 2000; van der Vegt and Janssen, 2003), may provide direct informational resources for IE. This aspect could be more crucial than demographic diversity, such as age, sex, and ethnicity (Miller et al., 1998). However, empirical studies on cognitive diversity yield mixed positive and negative results, necessitating further exploration from various perspectives (Mello and Rentsch, 2015).

Sociocultural backgrounds may influence the effectiveness of workplace diversity, and the Asia-Pacific context, including Japan, may exhibit unique features (Chen et al., 2023). In Japan, traditional business practices, such as lump-sum hiring of new graduates and lifetime employment, have fostered homogeneity in perceptions, knowledge, and skills among full-time Japanese male employees. While this approach has strengths like smooth decision-making and internal conflict resolution (Cabinet Office of Japan, 2019), it also results in a lack of awareness regarding the importance of workplace diversity in Japanese business organizations.

Despite this, the evolving business landscape emphasizes the necessity of diverse human resources for sustained innovation and growth in the face of increasing uncertainty (Ministry of Economy, Trade and Industry of Japan, 2018). Presently, academic research on workplace and cognitive diversity in Japan is insufficient in terms of quantity and content, with limited utilization of findings from overseas studies (Masaki, 2019). Therefore, this study conducted in Japan aims to deepen insights into cognitive diversity from a global perspective and provide implications for diversity management in the country.

Authentic leadership (AL) is a proven leadership theory positively influencing various team processes, including information processing (Alilyyani et al., 2018). AL can effectively promote IE and diversity management by fostering self-awareness, open communication, internalization of moral perspective, honest behavior, and continuous self-development among leaders (Avolio and Gardner, 2005; Gardner et al., 2005; Walumbwa et al., 2008). Authentic leaders also encourage followers to express their unique opinions and perspectives, fostering fruitful discussions and better decision-making, while promoting autonomous growth for both followers and the organization. Despite previous studies highlighting AL’s effectiveness on information processing, such as team reflexivity (Lyubovnikova et al., 2017), there has been a gap in examining AL’s impact on IE concerning workplace diversity.

**Figure 1:**
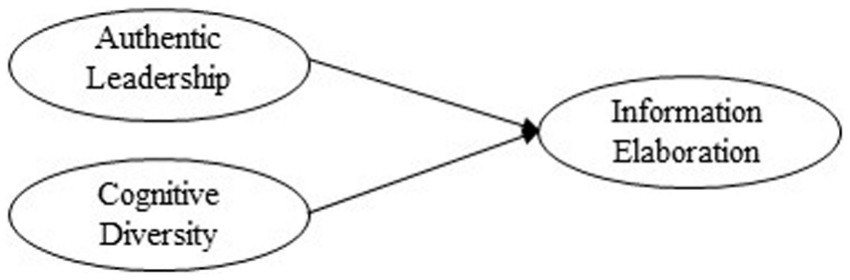
Conceptual framework.

Therefore, this study aimed to explore (1) the relationship between cognitive diversity and IE and (2) the effects of AL on IE within Japanese organizations, as illustrated in Figure 1.

## 2 Theory and hypotheses

### 2.1 Effect of information processing on team performance

Information processing within a team, involving the sharing, exchanging, discussion, and integration of task-relevant information, knowledge, ideas, expertise, insight, and perspectives, is imperative for realizing high-quality team performance, including creativity, innovation, and quality decision-making (van Knippenberg et al., 2004; Nonaka, 2007; Wang and Noe, 2010). For example, knowledge (Meng et al., 2016; Ul Hassan and Din, 2019) and information sharing promote employee creativity (Hahm, 2017). Furthermore, empirical research demonstrates the positive effects of knowledge sharing (Cheung et al., 2016) and information sharing (Moser et al., 2019) on team innovation. In summary, information processing within a team is essential for leveraging members’ diverse informational resources to achieve high team performance.

IE further elucidates the mechanism of information processing in attaining high team performance. The process of sharing information or knowledge, involving various steps where each team member engages in exchanging diverse perspectives, considering implications based on their unique expertise and viewpoint, communicating these implications to other members, and integrating them to produce optimal outcomes, collectively constitutes IE. This approach is most effective for performance excellence when a team task requires creativity and innovation, as opposed to simple and routine tasks (van Knippenberg et al., 2004).

Studies in various countries have highlighted the effectiveness of IE on several organizational outcomes. For example, it has been associated with team performance in Germany (Kearney and Gebert, 2009), team effectiveness and team viability in a global IT company in the US (Maynard et al., 2019), and creativity in Pakistan, China, and Taiwan (Lee and Yang, 2015; Oad and Niu, 2017; Wei et al., 2019). A meta-analysis by Roh et al. (2019) covering 51 studies in 10 countries (Belgium, Canada, China, India, Ireland, the Netherlands, Spain, Taiwan, the US, and the UK) found that information processing, such as IE, communication, and task-relevant debate among top management teams, positively influences organizational performance.

While not explicitly focusing on IE, Japanese studies have demonstrated the positive effects of equivalent team processes. Nawata et al.’s (2015) multilevel analysis revealed that team processes involving elaborative shared goals, achieving them, various task-relevant information, and frank and careful communication enhanced team performance. Similarly, Kimura (2016) explored various team learning processes and reported that reflective behavior, such as discussing team goals and outcomes, seeking feedback, and encouraging diverse and novel ways of thinking through trial and error, positively affected creative outcomes. The team processes investigated by Nawata et al. (2015) and Kimura (2016) encapsulate the core concept of IE. Therefore, both in Japan and overseas, IE may be crucial for achieving various positive team outcomes.

### 2.2 Cognitive diversity and information processing

Team members’ diversity serves as a crucial precursor in fostering IE to yield positive team outcomes, and cognitive diversity such as task-relevant information and the perspectives possessed by each member may have a more direct influence on IE (van Knippenberg et al., 2004). Based on the definitions such as Miller et al. (1998), Kilduff et al. (2000), and van der Vegt and Janssen (2003), we define cognitive diversity as the invisible cognitive differences of team members such as task-relevant knowledge, skills, ways of thinking, values, and so on, that can provide the resources for IE and lead to high team performance, such as creativity and innovation. Since the term “cognitive diversity” is used as a generic noun or as having various meanings in previous studies, our definition will be denoted as Cognitive DY. Instances where there is Cognitive DY among team members can lead to deeper reflections on these differences, more active discussions on the reasons and rationales behind them, and better integration of their diverse assets to arrive at improved conclusions. By contrast, it could also lead individuals to adhere rigidly to their own ideas and way of thinking, hindering open communication and impeding mutual understanding, thereby affecting overall team performance. Meanwhile, demographic diversity may or may not induce indirect effects through cognitive diversity (Miller et al., 1998). Even in demographically similar teams, significant cognitive differences can be perceived. Therefore, this study considered Cognitive DY as particularly valuable.

A meta-analysis conducted by Mello and Rentsch (2015) demonstrated that various aspects of cognitive diversity, including cognitive ability, values, and education, positively and negatively affect team processes and performances. Regarding IE effects, Hoever et al. (2012) found that the diversity of members’ perspectives benefited team creativity, mediated by IE, in a laboratory experiment. Shemla and Wegge (2019) found that the perceived educational diversity of team members positively affected IE. By contrast, perceived diversity in cultural attributes hindered communication openness, leading to negative effects on IE (Lu et al., 2017). While not specifically focused on cognitive diversity, a meta-analysis of task- and relations-oriented diversity by Roh et al. (2019) found positive effects on IE. Considering these varied results, it is significant to further examine how Cognitive DY affects IE.

### 2.3 Sociocultural characteristics and Japan’s current situation

Chen et al. (2023) posited that the consequences of workplace diversity might vary based on cultural backgrounds. In Japan, for example, several characteristics do not necessarily favor diversity. First, Japan is traditionally identified as having one of the most masculine cultures (Hofstede insights, n.d.), and the level of demographic diversity in business organizations in the country has been minimal, predominantly featuring Japanese male employees (Masaki and Muramoto, 2018). Regarding gender diversity, the female representation on the boards of publicly listed companies is 10.7%, significantly lower than in other advanced countries (Cabinet Office of Japan, 2021). Similarly, racial diversity is also low, with 97.5% being Japanese compared to 2.5% foreign workers (Cabinet Office of Japan, 2019).

Second, from an institutional perspective, Japanese business organizations have historically leaned toward homogeneity rather than diversity through specific Japanese employment practices, such as lump-sum hiring of new graduates, lifetime employment, and seniority-based systems (Cabinet Office of Japan, 2019). Third, these practices, in effect since the end of the 19th century (Cabinet Office of Japan, 2019), have a historical cultural basis in Confucianism, where social harmony, order, and respecting and obeying superiors are fundamental principles (Sai, 1991).

A cultural psychology theory elucidates why diversity may not align with Japanese business culture. Markus and Kitayama (1991) proposed that Asian cultures are characterized by interdependent self-construal, viewing the self as connected and less differentiated from others within the social context. By contrast, Western cultures exhibit independent self-construal, regarding the individual as independent and autonomous, with behavior organized primarily by internal references. Kitayama (1994) suggested several characteristics of interdependent self-construal that may affect diversity perceptions.

First, interdependent self-construal emphasizes self-restraint and maintaining harmony within the social context, while independent self-construal encourages uniqueness, self-expression, and valuing individual thoughts and feelings. Second, in interdependent self-construal, judgment is often aligned with one’s relationship with others, potentially forming similar judgment standards and reinforcing uniform thought processes as team tenure increases. Third, for interdependent self-construal, self-actualization involves being part of meaningful social relations rather than identifying one’s own internal valuable attributes. In summary, diversity may not be positively viewed in a culture of interdependent self-construal.

Based on these sociocultural and psychological characteristics, Japanese business organizations have traditionally consisted of highly homogeneous and competent employees, enabling efficient operations with minimal friction and internal conflicts, which has historically been the strength of Japanese companies (Cabinet Office of Japan, 2019). However, considering recent environmental changes such as continuous technological evolution, increased global competition, and growing complexity and uncertainty about the future, diversity, rather than homogeneity, is now deemed essential for business organizations to foster unprecedented innovation and survival (Ministry of Economy, Trade and Industry of Japan, 2018).

### 2.4 Cognitive diversity effect in the Japanese context

The aforementioned characteristics and situation in Japan suggest that Japanese society has not traditionally been pro-diversity. Here, diversity refers to diversity of any attributes including both demographic diversity and Cognitive DY. Given the awareness that diversity is more important than homogeneity for survival, as well as the global trend of promoting diversity, Ministry of Economy, Trade and Industry of Japan (2018) has advocated for promoting diversity management. Since Ministry of Economy, Trade and Industry of Japan (2018) has positioned the promotion of women’s advancement as the first step in diversity management, many companies have been first focusing on gender diversity and striving to increase the number of female employees and female managers. As a result, the inclusion of women has gradually been progressing in recent years, but the utilization of Cognitive DY of all employees, regardless of gender, is still in its infancy compared to Gender diversity (Cabinet Office of Japan, 2019).

This observation is evident in the results of international surveys and diversity studies. The survey on national culture conducted by the Global Leadership and Organizational Behavior Effectiveness (GLOBE) (2004) project revealed that Japan is the third highest among 62 countries that practice institutional collectivism, which is how organizational and societal institutional practices encourage and reward collective action. However, Japan ranked 57th for valuing institutional collectivism. Another notable finding of the GLOBE survey is that although Japan places the highest value on assertiveness among the other 62 countries, it ranks fourth lowest in assertiveness practice. These results indicate that the Japanese are still collectivistic regarding actual behavior, avoiding deviation from other members of their organizations but respecting uniqueness for values. Therefore, the behavior of leveraging Cognitive DY of team members and engaging in IE to improve team outcomes may not be well entrenched in the organization yet.

Few studies in Japan indicate the negative effects of diversity. Suzuki and Takemura (2016) found that differences in team members’ values and goals negatively affected social integration, leading to less creativity in new product development. Similarly, China has an obvious Confucian influence (Ohbuchi, 2015) and showed similar empirical findings regarding collectivism and assertiveness in the GLOBE survey (2004). Du (2016) found that Confucianism was negatively associated with board gender diversity. Chen et al. (2019) revealed that cognitive diversity negatively affected innovation work behavior mediated by relationship conflict. Lu et al. (2017) found that cultural cognitive diversity negatively affected IE mediated by communication openness.

Research on workplace diversity is sparse in Japan (Masaki, 2019), and studies on the relationship between specific diversity and information processing are further limited. Therefore, how Cognitive DY affects IE in Japanese business organizations must be examined. Based on the current business, social, and cultural context of Japan and theoretical and practical implications, we framed the following hypothesis.

*Hypothesis 1*: Cognitive DY negatively affects IE in Japan at both individual and team levels.

### 2.5 Authentic leadership and information elaboration

AL is one of the leadership theories demonstrated to positively affect various team processes and performance, including information processing (Alilyyani et al., 2018). AL is defined as a pattern of leader behavior based on deep self-awareness of one’s authentic beliefs, values, strengths, and weaknesses. It involves being authentic in a self-regulating manner, adapting to the situation and context of the team being led (Avolio and Gardner, 2005; Gardner et al., 2005; Walumbwa et al., 2008). Effective leaders behave authentically and continue to engage in self-development toward achieving their own and the team’s ultimate goals while respecting the authenticity of their team members, listening sincerely to their voices, and incorporating diverse perspectives to make the best decisions for the team. This attitude positively influences them, helping them grow together with the leader and achieve sustainable team growth (Gardner et al., 2005). Walumbwa et al. (2008) defined four dimensions of AL: (1) self-awareness (SA) — understanding one’s own values and characteristics and how they are derived, and deepening self-understanding by seeking feedback from others; (2) relational transparency (RT) — openly disclosing one’s authentic self to others to enhance mutual understanding and develop a trusting relationship; (3) balanced processing (BP) — objectively analyzing and soliciting relevant information before decision-making, even if it is against one’s own beliefs; and (4) internalized moral perspective (IM) —internalizing and integrating moral standards and values as the basis of self-regulated behavior without yielding to external pressure.

Empirical studies have shown the effectiveness of AL in promoting information processing, leading to positive team outcomes. Lyubovnikova et al. (2017) found that AL positively impacted team reflexivity, productivity, and effectiveness. Team reflexivity has a process similar to IE (Chen et al., 2019) and is defined as reflecting and communicating team objectives and processes (Lyubovnikova et al., 2017). Other AL effects are on information sharing (Hahm, 2017) and knowledge sharing (Meng et al., 2016; Ul Hassan and Din, 2019), both leading to employee creativity. Some studies examined the mediating variables between AL and knowledge sharing, such as team trusting atmosphere and psychological safety (Meng et al., 2016). Although not a direct examination of information processing, the mediating role of psychological safety in the relationship between AL and creativity has been found in several studies (Da Xu et al., 2017; Chaudhary and Panda, 2018). A multilevel analysis of small firm employees in the Netherlands, Poland, and Spain found that AL strengthened employees’ personal initiative and work engagement, leading to more innovative behavior (Laguna et al., 2019). Thus, AL may stimulate followers’ willingness and proactive action toward work goals. De Jong et al. (2018) examined the four AL dimensions in the Netherlands and India and found that BP contributed to open innovation in both countries.

Based on these findings, we suppose that all four AL dimensions play specific roles in promoting IE. As demonstrated by De Jong et al. (2018), BP utilizes various perspectives of diverse members to deepen the discussion. Moreover, when leaders solicitate opinions, even contrary to their own, a psychologically safe environment is fostered and team members will not hesitate to take a stance different from that of their superiors and express their thoughts and ideas. RT indicates leaders’ openness to proactively disclose their authentic selves and task-relevant knowledge and information. RT fosters an open, trusting, and psychologically safe environment, enabling team members to actively and frankly discuss with each other. Leaders’ IM, deeply rooted in their authentic selves, can consistently guide team processes to achieve the optimal outcome from both business and ethical perspectives. Lastly, SA involves a process of clarifying and deepening one’s own thoughts and beliefs (Walumbwa et al., 2008), and these authentic thoughts are the source of information to be shared with others and a driver of deep reflection in the IE process. AL is also effective from a theoretical perspective. IE may be hampered when diversity arouses social categorization, following the categorization and elaboration model (van Knippenberg et al., 2004). The aforementioned dimensions of AL promote self-respect for a person’s own authenticity, which generates respect for other’s authenticity and willingness to positively utilize diversity instead of categorizing and excluding different people. In summary, AL is expected to effectively promote IE within a team.

### 2.6 Authentic leadership effect in the Japanese context

In Japan, empirical research on AL is sparse, and the research setting is mainly limited to schools and higher education (Takenishi et al., 2013) instead of business organizations. Considering the current situation of the aforementioned Japanese business organizations, we assume that AL may positively promote IE by leveraging the Cognitive DY of team members.

First, AL can help mitigate Japanese people’s low assertiveness and reserved behavior. The discrepancy between the practice of assertiveness and the value placed on it, as revealed in the GLOBE study (2004), suggests that people understand the importance of assertion but hesitate to disclose their opinions openly. Furthermore, the Japanese tendency to value harmony based on Confucianism (Sai, 1991) may lead to non-assertiveness and avoidance of confrontation with others. However, when leaders disclose their authentic thoughts and encourage diverse perspectives, team members are encouraged to express their own unique creativity (Meng et al., 2016; Da Xu et al., 2017; Chaudhary and Panda, 2018). Second, the IM dimension in AL may be important for Japanese followers loyal to the top management due to the influence of Confucianism that suggests respecting elders (Sai, 1991). Japanese business managers, unlike those in other countries, view visionary and directive leaders as ethical and exemplary (Kimura and Nishikawa, 2018). Ishikawa (2009) reported that transformational leadership that influences subordinates with a clear vision and leads them toward higher performance through intellectual stimulation and individual consideration (Bass, 1999) significantly increased the consensus maintenance norm, suppressed followers’ open self-expressions, and reduced the quality of decision-making. These findings may indicate that if a leader sets an unethical example in Japan, there is a higher risk that the team members will follow without a thought. Therefore, it is essential for business organizations that leaders have internalized moral perspectives.

AL may affect both individual team members and the team as a whole. The authentic leader’s way of being and constant self-development serves as a role model for followers and inspires them to become authentic followers (Avolio et al., 2004; Gardner et al., 2005). In other words, AL has an impact at the individual level, as suggested by several studies (Hahm, 2017; Chaudhary and Panda, 2018; De Jong et al., 2018; Ul Hassan and Din, 2019). Simultaneously, AL encourages the development of authentic relationships between leaders and followers and creates an authentic organizational culture, which fosters positive behaviors and productive team processes throughout the team and enables positive organizational outcomes (Avolio et al., 2004; Gardner et al., 2005). This implies that AL has a team-level effect, as reported in several studies conducting team-level analyses (Edú-Valsania et al., 2016; Laguna et al., 2019). Therefore, we hypothesize that AL positively affects the IE at both individual and team levels.

*Hypothesis 2*: AL promotes the IE process at both individual and team levels.

## 3 Materials and methods

### 3.1 Participants and procedures

Considering that Japanese companies, regardless of industry sector or size, are encouraged to promote diversity, and all companies must transform themselves through innovation for sustainable growth (Ministry of Economy, Trade and Industry of Japan, 2018), this study collected data from several industries. Moreover, we established the inclusion criteria considering that multiple team members and leaders engaged in IE are essential to its discussion, and invited participants who met these criteria. Specifically, the study data were collected from several industries and organizations from June to August 2020 using the snowball sampling approach. We sent invitation letters explaining the research purpose and inclusion criteria for the participating team to 432 persons from more than 400 organizations via e-mail or equivalent electronic forms. The inclusion criteria were a team comprising three or more members and one leader with command over the members and the ability to enhance communication within the team to discuss tasks and processes daily for achieving team objectives. We received valid responses from 111 teams (25.7% response rate) after excluding incomplete answers. Subsequently, we excluded 21 teams with less than three members, which was required to examine the team processes with multilevel analysis (e.g., Wang et al., 2016; Chow, 2018). Thus, the final sample comprised 375 participants from 90 teams from 66 organizations.

Of the participants, 57.3% were male, 40.8% were female, 0.3% reported other gender, and 1.6% provided no response. Regarding age, 3.5% were younger than 25 years old, 29.1% were 25–35 years old, 36.5% were 35–45 years old, 23.7% were 45–55 years old, 5.3% were older than 55 years, and 1.9% provided no age data. Regarding education, 6.4% had graduated from junior high or high school, 9.1% from business or technical college, 4.5% from junior college, 57.6% from university, and 19.5% from graduate school. Regarding the team tenure of each member, 17.6% were for less than a year, 37.9% for 1–3 years, 26.7% for 3–10 years, 12.3% for 10–20 years, and 2.7% for more than 20 years. The average team size was 4.17 members (ranging from 3–8). Regarding the industry type of 90 teams, 28.9% of teams were service business, 22.2% from manufacturing, 10.0% from the information technology (IT) industry, 8.9% from finance or insurance, 6.7% from retail or wholesale, 4.4% from public service, and 18.9% from other industries.

We used Google Forms to conduct the online survey, which allowed us to set up a unique URL for each team, enabling us to identify who belonged to which team. In creating the survey, we were sensitive to the possible influences of social desirability response biases — the tendency of participants to provide socially desirable answers to self-report questionnaires (Randall and Fernandes, 1991) and common method biases — the bias caused by the measurement method rather than by the constructs that the measures represent (Podsakoff et al., 2003). To avoid social desirability response biases, the following points were mentioned on the cover page of the survey: (1) the survey is conducted purely for academic purposes and is not affiliated with your organization, and participation is not compulsory; (2) all of participants can skip a question if they do not want to answer or stop the survey immediately, without any disadvantage, including but not limited to personnel evaluation in your organization; (3) the survey data is handled anonymously and thus your answers will never be disclosed to anyone and be used solely for research purposes. To avoid common method bias, the following measures were taken: (1) the questions of the independent and dependent variables were randomly placed in sequence to make them appear unrelated; (2) it was ensured that the questions were simple and specific by revising the wordings through a pilot test among working persons; and (3) the names of variables were omitted to reduce unnecessary guessing.

We sent the URL information to team representatives who confirmed study participation in response to the invitation letter and requested them to forward the URL to the team members who were expected to participate. The participants’ answers were automatically collected on the Internet as a team. Finally, 375 participants from 90 teams completed the questionnaires after providing informed consent.

Before starting the study, ethical approval was obtained from the Human Research Ethics Committee of the researchers’ university.

### 3.2 Measures

As the scales for assessing AL and IE were in English, we translated them to Japanese in accordance with the guidelines provided by the International Society for Pharmacoeconomics and Outcomes Research task force (Wild et al., 2005). With permission from the original authors, the scales were translated to Japanese by two professionals, then back-translated to English by a bilingual professional translator and a professor who specializes in psychology at the university. The back-translated English versions were verified and approved by the original authors.

To examine the clarity of the questions in the translated version, we recruited collaborators through the affinity method and obtained informed consent from 10 working adults. The demographic characteristics of the collaborators were: five males and five females; five were 25–35 years old, four were 35–45 years old, and one was between the age of 45–55 years. We sent them the URL of the Google Form and requested them to answer the questions and comment on the questionnaire. The collected comments were utilized to revise the Japanese version further.

#### 3.2.1 Cognitive diversity

This study referred to van der van der Vegt and Janssen’s (2003) scale, which addressed by measuring team members’ differences in their ways of thinking, in their knowledge and skills, in how they viewed the world, and in their beliefs about what is right and wrong. This scale was widely used by previous studies that examined the relation between cognitive diversity and IE and relevant team processes (Kearney and Gebert, 2009; Wang et al., 2016; Chow, 2018; Chen et al., 2019). In addition, since this scale fits reasonably well with the definition of Cognitive DY mentioned in Section 2.2 above, we based our development of an eight-item measure on van der Vegt and Janssen’s (2003) scale. Our measure consists of two dimensions: cognitive differences between oneself and other team members (self-to-others DY) and those among team members (members DY). Each dimension had four items to assess respondents’ perspectives, knowledge and skills, worldviews, and beliefs about right and wrong. Sample items for self-to-others DY include “To what extent do you differ from other team members in your way of thinking?” and for members DY include “To what extent do the team members differ from each other in their knowledge and skills?” Responses were based on a five-point Likert scale ranging from 1 (a minimal extent) to 5 (a considerable extent). The Cronbach’s α of both self-to-others DY and members DY were 0.72.

#### 3.2.2 Authentic leadership

We used the Japanese translation of the 16-item Authentic Leadership Questionnaire (ALQ) by Walumbwa et al. (2008). ALQ consists of four substantive factors: relational transparency (AL-RT, five items), internalized moral perspective (AL-IM, four items), balanced processing (AL-BP, three items), and self-awareness (AL-SA, four items). Sample items include “my leader says exactly what they mean” (AL-RT) and “my leader makes decisions based on their core values” (AL-IM). Responses were based on a five-point Likert scale ranging from 1 (not at all) to 5 (frequently, if not always).

To confirm whether the same factor structure applied to the Japanese version of the ALQ, a confirmatory factor analysis was conducted. The results showed a slightly poor fit (*χ^2^* (98) = 447.317, *p* = 0.000, CMIN/DF = 4.564, comparative fit index [CFI] = 0.896, incremental fit index [IFI] = 0.897, root mean square approximation error [RMSEA] = 0.103). The standardized coefficient was 0.194 for “My leader displays emotions exactly in line with feelings,” which was notably low among the five questions on AL-RT. AL-RT refers to building transparent relationships with others and promoting trust by disclosing actual thoughts and feelings to others (Walumbwa et al., 2008). This item is related to expressing subjective emotions, while the remaining four are related to disclosing concrete opinions and facts. It is characteristic of the traditional Japanese male not to express emotions (Watanabe, 2017). According to Taga (2006), Tanaka (2009), and Watanabe (2019) there are many male-dominated organizations in Japan, and men are still expected to play traditional masculine roles. As the leaders in this study were predominantly male and relatively old (73% male, median age between 45 and 55 years old), their subordinates may have expected their leaders’ traditional behavior of suppressing emotions, explaining why the responses for this item differed from the other four items. We contacted the original authors and received approval for deleting this item due to cross-cultural issues. A confirmatory factor analysis without this item revealed improved fit indices [*χ^2^*(84) =349.984, *p* = 0.000, CMIN/DF = 4.166, CFI = 0.919, IFI = 0.919, RMSEA = 0.096]. As in the original study, the other subfactors were measured with four items for AL-IM, three for AL-BP, and four for AL-SA. The Cronbach’s α were 0.81, 0.83, 0.83, and 0.87 for AL-RT, AL-IM,. AL-BP, and AL-SA, respectively.

#### 3.2.3 Information elaboration

We used the Japanese translation of the seven-item IE scale by van Dick et al. (2008). Sample items include “My team members exchanged a lot of information about the task” and “In my team, we discuss the content of our work a lot.” Responses were based on a five-point Likert scale ranging from 1 (totally not applicable) to 5 (totally applicable). The Cronbach’s α was 0.89.

#### 3.2.4 Control variables

This study included several control variables. Following previous research (Wang et al., 2016; van Zijl et al., 2019), we controlled for age to test the effects of Cognitive DY over and beyond demographic diversity. We also controlled for team size, which influences team processes such as IE. Age was coded as 1 = younger than 25 years old, 2 = 25–35 years old, 3 = 35–45 years old, 4 = 45–55 years old, and 5 = older than 55 years based on prior research (Nohe et al., 2013; Shen et al., 2014). Team size was coded as 1 = less than 4 members, 2 = 4–10 members, 3 = 10–20 members, and 4 = more than 20 members.

### 3.3 Data analysis

Our data contained a hierarchical structure, wherein responses of individual-level variables were nested within teams. From a theoretical perspective, AL, in particular, impacts individual team members and the team as a whole. Therefore, we conducted a multilevel analysis using multilevel structural equation modeling (MSEM) to test the hypotheses using R4.1.2 (lavaan package 0.6–9) based on Preacher et al. (2010). Missing data were handled using the complete information maximum likelihood and maximum likelihood estimation as estimators. To examine whether the study variables would be appropriate to aggregate individual responses at the team level, we calculated ICC(1), ICC(2), and rwg. ICC(1) indicates how individuals within the same team agree in their perceptions of the team characteristics. It is recommended to be higher than 0.05 with a median of 0.12 for aggregation (James, 1982). ICC(2) indicates reliability of the means at the aggregate level and is insufficient for aggregation if less than 0.50 (Ostroff, 1992; Xu and Hou, 2018). The rwg score is an indicator of the agreement between team members and is calculated using the difference between the observed variance and theoretical variance, which is expected when there is no agreement at all, and above 0.70 is empirically approximate for data aggregation (James et al., 1984; Suzuki and Kitai, 2005).

The goodness of fit of the model was evaluated based on the CFI and RMSEA. For the CFI, values above 0.90 were considered to indicate an acceptable fit (Marsh and Hau, 1996; Salisbury et al., 2002). For the RMSEA, values in the range of 0.05–0.08 were defined as fair fit and 0.08–0.10 as mediocre fit (MacCallum et al., 1996).

## 4 Results

### 4.1 Descriptive statistics and data aggregation

Table 1 presents descriptive statistics and intraclass correlation coefficients (ICC): ICC(1), ICC(2), and rwg at the individual level. The reliabilities of all the variables were acceptable.

**Table 1:** Descriptive statistics and ICC (Individual level).

		*M*	*SD*	*α*	ICC(1)	ICC(2)	*rwg*
Diversity	Self-to-others	2.41	0.65	0.72	0.10	0.30	0.82
	Members	2.48	0.59	0.72	0.06	0.21	0.83
Authentic leadership	Relational transparency	3.68	0.79	0.81	0.21	0.53	0.76
	Internalized moral perspective	3.42	0.82	0.83	0.28	0.60	0.75
	Balanced processing	3.37	0.87	0.83	0.21	0.51	0.71
	Self-awareness	3.05	0.89	0.87	0.24	0.55	0.69
Information elaboration		3.09	0.75	0.89	0.20	0.51	0.78

*n* = 375.

Although our results show that only ICC(2) scores of self-to-other DY and members DY did not exceed the recommended level, other scores of these variables were sufficient for aggregation. Therefore, we considered the aggregate of all variables at the team levels.

The individual responses of the subfactors of self-to-others DY, members DY, AL, and IE were aggregated to the team-level; the resultant descriptive statistics of these team level variables are shown in Table 2.

**Table 2:** Descriptive statistics (Team level).

		*M*	*SD*	*α*
Diversity	Self-to-others	2.42	0.39	0.75
	Members	2.47	0.34	0.69
Authentic leadership	Relational transparency	3.70	0.49	0.82
	Internalized moral perspective	3.43	0.54	0.87
	Balanced processing	3.40	0.55	0.86
	Self-awareness	3.05	0.59	0.90
Information elaboration		3.11	0.47	0.91

*n* = 90.

Table 3 shows the correlations for all variables at the individual level. Statistically significant correlations were observed between self-to-others DY and members DY, four subfactors of AL, and IE. Regarding the control variables, age was negatively associated with most other variables, except for team size and members DY, while team size did not have any significant relations with other variables.

**Table 3:** Correlation matrix for study variables (Individual level, raw scores).

			1	2	3	4	5	6	7	8	9
1	Age		1.00								
2	Team size		0.01	1.00							
3	Diversity	Self-to-others	−0.13*	0.00	1.00						
4		Members	−0.07	−0.01	0.56**	1.00					
5	Authentic leadership	Relational transparency	−0.15**	−0.05	−0.16**	−0.14**	1.00				
6		Internalized moral perspective	−0.12*	−0.03	−0.13*	−0.13*	0.77**	1.00			
7		Balanced processing	−0.15**	0.00	−0.13*	−0.13*	0.66**	0.71**	1.00		
8		Self-awareness	−0.16**	−0.03	−0.17**	−0.12*	0.68**	0.74**	0.71**	1.00	
9	Information elaboration		−0.21**	0.05	−0.18**	−0.11*	0.49**	0.44**	0.44**	0.42**	1.00

*p = 0.05, **p = 0.01.

### 4.2 Hypotheses testing

**Figure 2:**
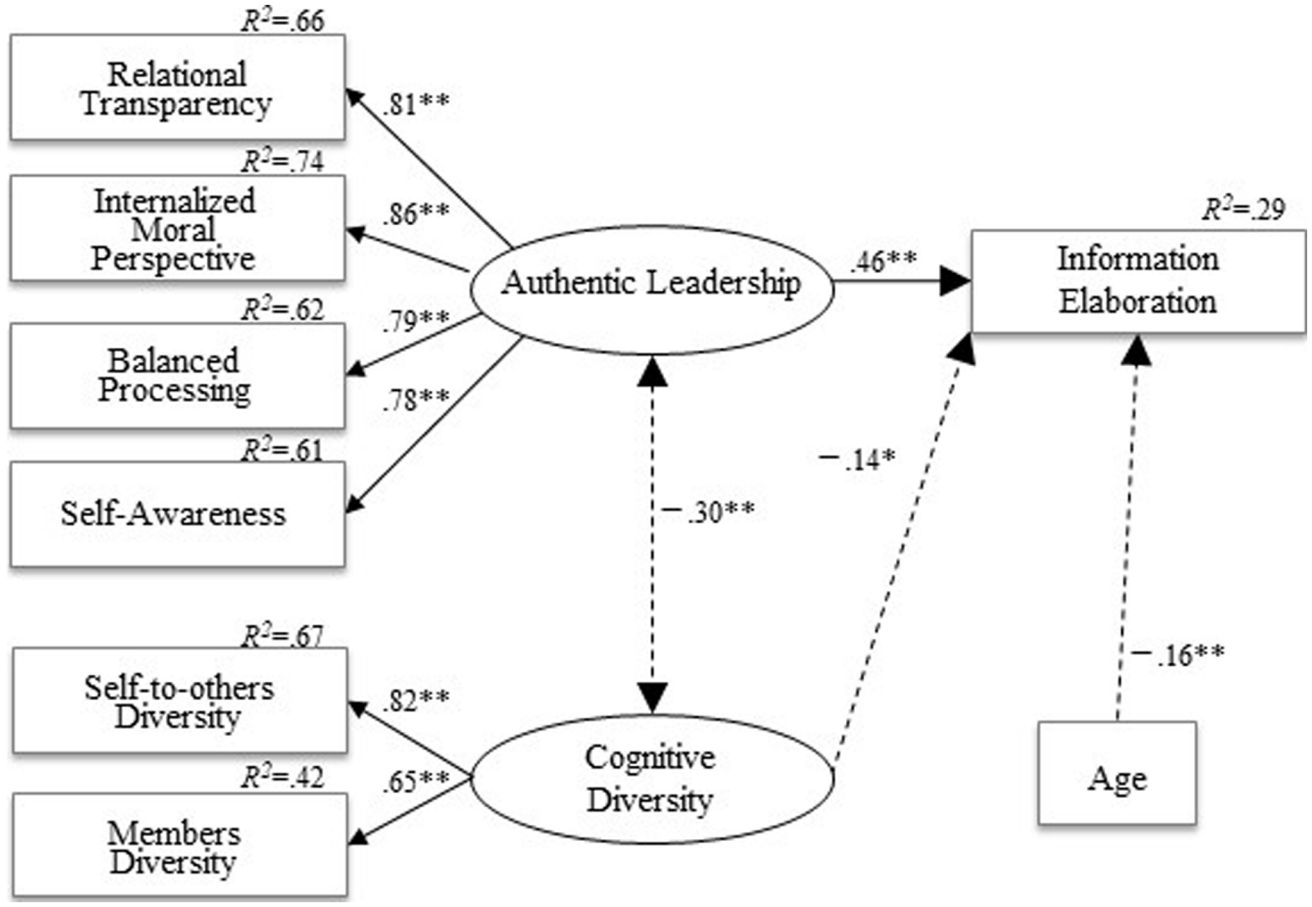
Individual level structural equation model. Only statistically significant paths are shown. Standardized coefficients appear on single-headed arrows. Covariance appears on double-headed arrows. Positive coefficients appear in bold lines, while negative coefficients appear in dotted lines.

**Figure 3:**
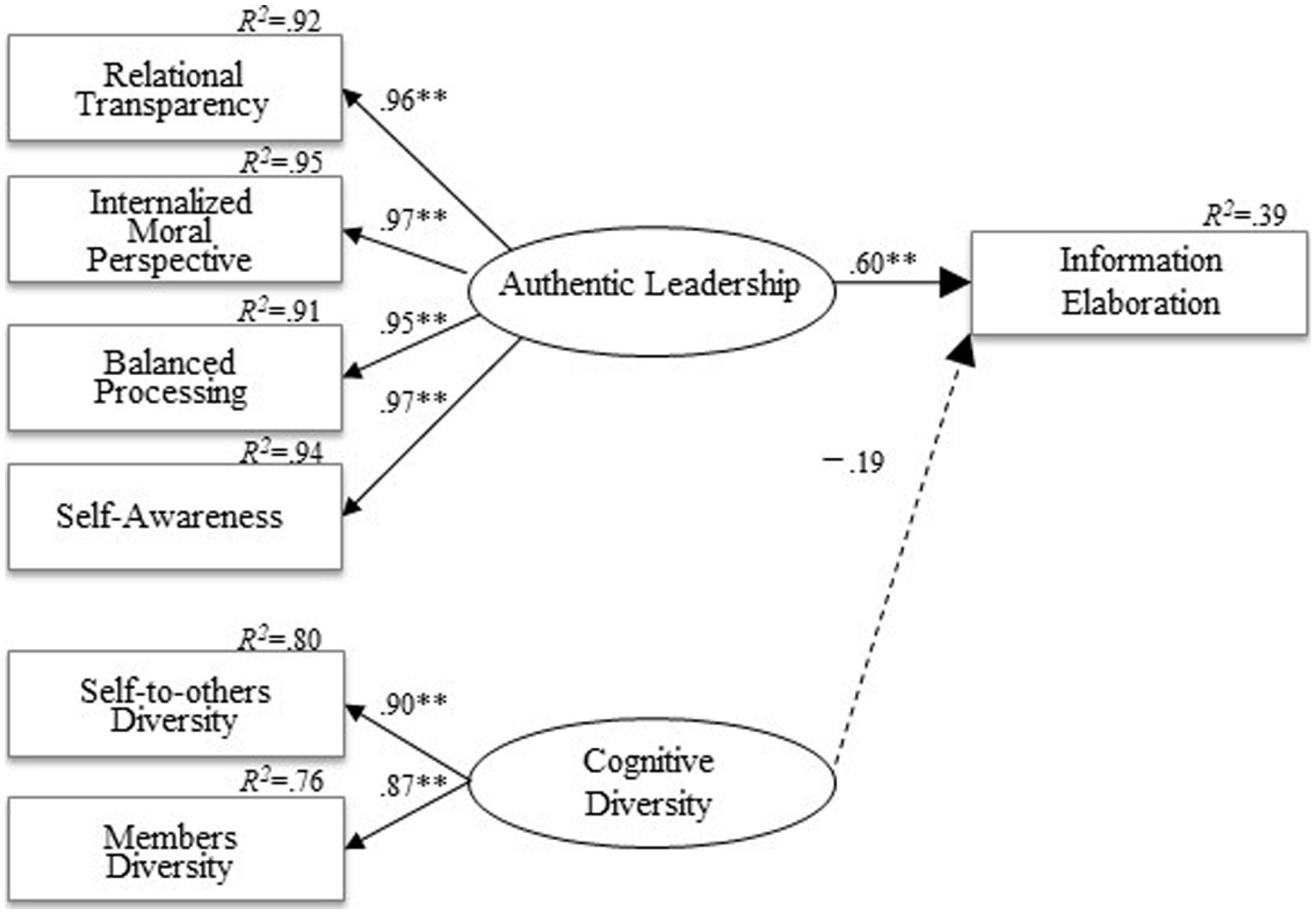
Team level structural equation model. Only statistically significant paths are shown. Standardized coefficients appear on single-headed arrows. Covariance appears on double-headed arrows. Positive coefficients appear in bold lines, while negative coefficients appear in dotted lines.

To test Hypotheses 1 and 2, we conducted MSEM analysis using R4.1.2. (lavaan package 0.6–9). The analysis model was set up based on the hypothesized model shown in Figures 2, 3, and two latent variables were expected. The first was AL, consisting of four substantive factors: AL-RT, AL-IM, AL-BP, and AL-SA. The second latent variable was Cognitive DY, consisting of self-to-others DY and members DY. As shown in Figures 2, 3, factor loadings of the two latent variables were acceptable. Age and team size were also included in the model at the individual level as controlling factors.

The fit indices of the resulting model were acceptable [χ2 (41) = 66.182, *p* = 0.000, CMIN/DF = 1.614, CFI = 0.978, IFI = 0.978, RMSEA = 0.041, Akaike information criterion [AIC] = 4481.136, Standardized Root Mean Squared Residual [SRMR] (within) = 0.049, SRMR (between) = 0.089].

Hypothesis 1 predicted that Cognitive DY negatively affects IE at both individual and team levels. First, at the individual (within) level, Cognitive DY negatively affected IE (*β* = −0.14, *B* = −0.18, SE = 0.09, *p* = 0.047). Individual members perceived a high dissimilarity among members within their team, which hindered the team process of open opinion exchanges and elaborate discussion for better decision-making or problem-solving. This result is consistent with the negative correlation between Cognitive DY and IE shown in Table 3. Therefore, Hypothesis 1 at the individual (within) level was supported.

Next, at the team (between) level, the effect of Cognitive DY on IE was not significant, though negative (*β* = −0.19, *B* = −0.30, SE = 0.39, *p* = n.s.). Therefore, Hypothesis 1 at the team (between) level was not supported. Compiling both the individual and team levels, Hypothesis 1 was partially supported.

Hypothesis 2 predicted that AL promotes IE at both individual and team levels. First, at the individual (within) level, AL positively affected IE (*β* = 0.46, *B* = 0.51, SE = 0.07, *p* < 0.01). This finding indicates that when the individual member perceives that their leader is authentic, they feel inspired toward new learning, deep thinking, and novel ideas, and actively discuss task-related matters.

Next, at the team (between) level, AL also positively affected IE (*β* = 0.60, *B* = 0.45, SE = 0.14, *p* < 0.01). In sum, a team led by an authentic leader is actively engaged in the IE process. Therefore, Hypothesis 2 was supported.

Although it was not hypothesized, the correlation between Cognitive DY and AL was significantly negative at the individual level (*β* = −0.30, *B* = −0.09, SE = 0.02, *p* < 0.01).

## 5 Discussion

This study examined two hypotheses. The first hypothesis was that Cognitive DY negatively affects IE in Japanese business organizations at both individual and team levels, considering the Japanese sociocultural and organizational context, which is traditionally homogeneous rather than diverse and respects harmony. The results partially supported the hypothesis. At the individual level, Cognitive DY negatively affected IE. This result is consistent with empirical research in China (Du, 2016; Lu et al., 2017; Chen et al., 2019), which has a similar sociocultural background to Japan, such as Confucianism and interdependent self-construal. In contrast, the effect of Cognitive DY on IE was not significant, though negative at the team level. It implies that perceptions of cognitive differences among team members may vary within a team.

The second hypothesis was that AL positively affects IE at individual and team levels, which was fully supported. Previous studies have demonstrated that AL positively impacts team processes, such as information sharing (Hahm, 2017) and knowledge sharing (Ul Hassan and Din, 2019). However, the effect on IE, a specific team process that further elaborates and clarifies how information processing takes complete advantage of diversity, was not examined (van Knippenberg et al., 2004). Therefore, this study’s findings confirmed that AL is effective for diversity-related team processes and positively affects informational processing.

### 5.1 Theoretical implications

#### 5.1.1 Negative effect of cognitive diversity on information elaboration in Japanese organizational context

This study’s primary contribution is further clarifying the effect of Cognitive DY on IE in the Japanese context, alongside various research in other countries. IE is generally enhanced by cognitive diversity (Hoever et al., 2012; Shemla and Wegge, 2019), although diversity can positively or negatively affect team performance (van Knippenberg et al., 2004).

First, at the individual level, we found that Cognitive DY negatively affected IE. This finding reflects the Japanese sociocultural and organizational context, which is traditionally homogeneous and respects harmony due to Confucianism and interdependent self-construal. There are several alternative possible explanations for this finding. First, the nature of team tasks in the Japanese organizational context may differ from the proposed relationship between diversity and IE. van Knippenberg et al. (2004) proposed that diversity may benefit performance more effectively when a team task requires information processing for creative and innovative solutions in an organizational context rather than simple and routine tasks. Indeed, some studies examining the effect of IE deliberately selected specific teams from a few companies, wherein IE was likely to be more critical to executing tasks, such as research and development teams in multinational pharmaceutical companies (Kearney and Gebert, 2009; Wang et al., 2016) and global virtual teams of an international IT company (Maynard et al., 2019). Wei et al. (2019) invited participants from the manufacturing, communications, energy, and finance industries because these industries are under significant pressure to innovate. As this study did not focus on any specific industry where innovation is mandatory, it is possible that some participants or teams were not engaged in creative and innovative tasks. Based on the classification in the study by Wei et al. (2019), we conducted a correlation analysis by extracting data only from manufacturing, IT, and financial industries and observed that the correlations between Cognitive DY on IE continued to be negative (self-to-other DY: *β* = −0.18, *p* < 0.05; members DY: *β* = −0.13, *p* = n.s.). Therefore, the negative effects of Cognitive DY on IE may be from Japanese sociocultural characteristics. The tendencies of Japanese workplaces to value harmony (Sai, 1991) and avoid assertive expression of diverse opinions (Global Leadership and Organizational Behavior Effectiveness (GLOBE), 2004) may prevent IE, despite diverse opinions being beneficial in stimulating team creativity and innovation.

Another possible explanation could be that Cognitive DY aroused team conflict, negatively affecting team performance (Chen et al., 2019). van Knippenberg et al. (2004) proposed that any dimension of diversity may be the source of social categorization, eliciting relational conflict and causing a disruptive effect on IE. In this study, such a mediation effect of team conflict might have worked between Cognitive DY and IE. To evaluate this possibility, we conducted another analysis for task interdependency, assuming that participants mainly engaged in independent tasks would have less opportunity to interact with other members and, therefore, have fewer conflicts. However, the result showed significant negative effects of Cognitive DY on IE, even for those mainly engaged in independent tasks. Although this finding cannot completely dismiss the possibility that conflict mediated the relation between Cognitive DY and IE, it cannot be interpreted solely considering this mediating effect, and factors such as Japanese sociocultural characteristics may have played a certain role.

Second, at the team level, the negative effect of Cognitive DY on IE was not significant, as opposed to the negative effect at the individual level. This finding suggests a new tendency that Japanese organizations are no longer as homogeneous as previously practiced. The traditional Japanese corporate culture emphasizes harmony, a homogenous way of thinking, and sharing the same values. The previous generations lived with such social beliefs that negatively perceived Cognitive DY, whereas the younger generation may be more respectful of each person’s individuality and view Cognitive DY positively. Consequently, the negativity against Cognitive DY may not have been high enough to be significant, which may also be valid for the result that the covariance between Cognitive DY and AL was not significant at the team level. Indeed, there are differences in perceptions of Japanese employment practices among generations. For instance, more people in the 50s generation believed that the seniority system had both advantages and disadvantages than those who believed it had only disadvantages, whereas the ratio was reversed for those in their 40s and younger (Cabinet Office of Japan, 2019). Meanwhile, more than 60% of all generations responded that workplace diversity had more advantages than disadvantages (Cabinet Office of Japan, 2019). These findings suggest that, although the generation gap still somewhat exists, the older generation has begun to understand the need for diversity as the future direction.

As this study was the first in Japan to examine the effect of Cognitive DY on team information processing, we did not investigate intergenerational differences in Cognitive DY. Future studies must examine whether and how much Cognitive DY varies among different generations for further insights.

#### 5.1.2 Positive effect of authentic leadership on information elaboration

Another contribution of our study lies in examining the effectiveness of AL as a leadership to promote IE at both individual and team levels. While the positive effect of transformational leadership on the relationship between workplace diversity and team performance has already been examined (Kearney and Gebert, 2009), this study confirmed that AL positively affected IE at both individual and team levels. This indicates that the leader’s demonstration of AL can influence individual members to actively express their own honest opinions, to have open discussions with each other, and become proactively engaged in the IE process, and for the team atmosphere to proactively undertake the IE process.

The influence of AL on IE may have been mediated by other variables. Previous studies have presented several mediating variables between AL and team outcomes, such as team climate of trust and psychological safety (Meng et al., 2016). Indeed, leadership, team climate, and team processes may interplay with each other (Wang and Noe, 2010; Guillaume et al., 2017). Although this study did not investigate any mediating variables between AL and IE, some other variables might have played a mediating role. Future studies must investigate such a mediating mechanism.

Another contribution of this study lies in advancing the literature toward verifying the effect of AL in the Japanese business context for the first time. Japanese organizations are traditionally homogeneous (Masaki and Muramoto, 2018) and do not necessarily have a solid sociocultural background to embrace diversity, as they place more value on relationships among team members than on the uniqueness of individual members due to interdependent self-construal (Markus and Kitayama, 1991). Such Japanese characteristics may not necessarily be compatible with the premise of AL, which is awareness of one’s authenticity, open disclosure, and respect for the uniqueness of others. However, the result that AL positively impacted team processes in Japan indicates that AL is universally applicable across countries, regardless of varied sociocultural contexts.

Although it was not hypothesized, the correlation between Cognitive DY and AL was small but significantly negative. This result may be explained by the socio-cultural and organizational background of Japan as discussed in Section 5.1.1. In the culture that traditionally values homogeneity and harmony, it is possible that the lower the Cognitive DY among members, the more a sense of unity is felt rather than the other way around, and therefore, the leader may be able to behave in an authentic manner. In such an organization, if activities focused solely on enhancing Cognitive DY are promoted, AL may be weakened and, in turn, may not be as effective enough to facilitate IE as shown in this study.

### 5.2 Practical implications

This study’s findings offer several implications for diversity management. First, although workplace diversity has generally been encouraged worldwide to generate positive business outcomes, such as creativity and innovation, Cognitive DY can negatively affect business organizations that are highly homogeneous and traditionally do not value diversity (Masaki and Muramoto, 2018). Therefore, to ensure diversity management success in such organizations, all team members must respect their diverse thoughts, ideas, perspectives, knowledge, and skills, that is, Cognitive DY, and fully utilize them in elaborating discussions for better team outcomes. Once individual members share the benefit of Cognitive DY, their tendency toward homogeneous thinking and harmonious behavior could help individuals engage in IE with others, and such individual behavior can be realized at the team level, ultimately enabling the company-wise deployment acceleration of diversity management.

Second, our findings of the effectiveness of AL on IE provide the direction of leadership development in implementing diversity management, especially in traditionally homogeneous organizations.

Japanese organizations have been demographically homogeneous; however, the cognitive aspects of individual members, especially the information, personal opinions and ideas, and perceptions held by each individual, may differ. When leaders recognize that each team member is unique regardless of one’s demography, respect and encourage to express one’s authentic self and advocate that utilizing diverse perspectives of team members is somewhat helpful to team performance (Avolio and Gardner, 2005; Gardner et al., 2005; Walumbwa et al., 2008), members gain confidence in their own uniqueness, which is different from others, and become motivated to utilize their unique abilities actively.

Leaders’ authentic attitudes can mitigate the negative aspect of interdependent self-construal that values harmony with others at the cost of actual self-expression (Kitayama, 1994) and the tendency to silently obey superiors without asserting oneself (Sai, 1991). When promoting diversity management in an organization that is traditionally homogeneous, it is essential to balance the enhancement of members’ Cognitive DY and AL at the same time, and it can be effective to provide leader training to foster AL. In a highly dynamic business environment that is becoming more global and diverse and requires constant innovation for sustainable growth, AL can catalyze utilizing the diverse potential of employees in all industries and countries to continue enhancing innovation.

### 5.3 Limitations and future research directions

This study has some limitations. First, regarding data collection, the cross-sectional research design made it challenging to conclude causal relationships. Additionally, the self-reported data may have generated common method variance bias, although we took measures to prevent it when developing the questionnaires. Future research should consider using a longitudinal design with specific time gaps between assessing variables and collecting information from separate sources.

Second, this study emphasized the effect of Cognitive DY on IE in traditionally homogeneous organizations that may not have a positive view of leveraging diversity for better team outcomes, as well as the effect of AL, which respects and leverages members’ diversity, on IE. As a result, we did not consider the possible mediating variables between Cognitive DY or AL and IE. Negative effects on IE may have been mediated by some variables that generate social categorization and team conflicts, as mentioned earlier. Existing empirical studies have examined IE and social categorization as a separate team process that affects team performance (Chen et al., 2019). It will be valuable to comprehensively investigate the relationship between IE and social categorization through an integrated analysis of both processes.

Third, in examining organizations with sociocultural backgrounds characterized as traditionally homogeneous and interdependent self-construal, this study analyzed only data from Japanese organizations. Future research should conduct detailed comparative investigations of the differences among countries with different social, cultural, and organizational backgrounds.

## Data availability statement

The raw data supporting the conclusions of this article will be made available by the authors, without undue reservation.

## Ethics statement

The studies involving humans were approved by the Human Research Ethics Committee of the University of Tsukuba. The studies were conducted in accordance with the local legislation and institutional requirements. The participants provided their written informed consent to participate in this study.

## Author contributions

KY: Writing – original draft. JI: Writing – review & editing. KF: Writing – review & editing.
